# U.S. Army Mortality Surveillance in Active Duty Soldiers, 2014–2019

**Published:** 2024-05-20

**Authors:** Gabrielle F. Kaplansky, Maisha Toussaint

**Affiliations:** 1 General Dynamics Information Technology Inc., Falls Church, VA; 2 Division of Behavioral and Social Health Outcomes Practice, Defense Centers for Public Health–Aberdeen, Defense Health Agency, Aberdeen Proving Ground, MD

## Abstract

Mortality surveillance is an important activity for capturing information on a population’s health. This retrospective surveillance analysis utilizes administrative data sources to describe active duty U.S. Army soldiers who died from 2014 to 2019, and calculate mortality rates, assess trends by category of death, and identify leading causes of death within subpopulations. During the surveillance period, 2,530 soldier deaths were reported. The highest crude mortality rates observed during the 6-year surveillance period were for deaths by suicide, followed by accidental (i.e., unintentional injury) deaths. The crude mortality rates for natural deaths decreased significantly over the 6-year period, by an average of 6% annually. The leading causes of death were suicide by gunshot wound, motor vehicle accidents, suicide by hanging, neoplasms, and cardiovascular events. Significant differences were observed in the leading causes of death in relation to demographic characteristics, which has important implications for the development of focused educational campaigns to improve health behaviors and safe driving habits. Current public health programs to prevent suicide should be evaluated, with new approaches for firearm safety considered.

**What are the new findings?:**

The mortality rate for natural causes declined 6% annually, from 18.8 deaths per 100,000 soldiers in 2014 to 13.4 deaths per 100,000 in 2019, which was statistically significant. During this period, when annual mortality rates for natural deaths declined significantly, the highest Army mortality rates were for deaths due to suicide, followed by accidental death. Despite the decline in natural deaths, neoplasms remain the leading cause of death in women and older soldiers.

**What is the impact on readiness and force health protection?:**

This report provides more accurate mortality surveillance for the Army population and is the only all-cause mortality report published by the Defense Health Agency since 2016. Preventable deaths are a significant issue in the Army population. A better understanding of these deaths can focus attention on both behavioral and medical factors that affect military readiness. This report reveals trends in mortality and related subject areas that require more active or renewed prevention efforts.

## 1 BACKGROUND

Mortality surveillance is an important activity for capturing information on a population’s health, as it tracks new and emerging health trends in a population and informs future prevention efforts.^1^ Mortality surveillance in the U.S. Army is essential for identifying and understanding the occupational exposures that increase risk of premature soldier death.^2^ Given that approximately 70% of soldiers are young adults under 35 years of age, this translates to significant potential years of life lost.

Few public health investigations have focused on all-cause mortality in the U.S. Armed Forces.^2,3,4^ Prior investigations within the military were restricted to specific categories and causes of death, such as neoplasms, infectious diseases, and suicide.^5,6,7,8^ The few investigations that examined all-cause mortality concluded that male, non-Hispanic White, and 17-34-year-old service members had the highest mortality rates in the U.S. military.

No known prior studies have examined the differences in the leading causes of death among subpopulations, such as sex, age, and racial ethnicity, in the U.S. Army. Strata-specific analysis by demographic characteristics is an important epidemiological methodology that recognizes consequential social, environmental, and biological differences among subgroups.^9^ The objectives of this study were to describe the demographic characteristics of U.S. Army active duty soldiers who died from 2014 to 2019, identify leading causes of death within subpopulations, and calculate mortality rates to assess trends by category of death.

## 2 METHODS

**Study Design and Population**

This retrospective surveillance analysis included information on mortality among U.S. Army active duty (Army active component, activated National Guard or Reserve) soldiers from 2014 to 2019. Soldiers who were between 17 and 64 years of age at the time of their death were included in this study. This project was reviewed and approved by the Office of Human Protections Public Health Review Board, Defense Centers for Public Health–Aberdeen.

**Data Sources and Study Variables**

The Defense Casualty Information Processing System (DCIPS), which collects information on service members who die while in service, was the primary source of category of death, as its data are more complete. If information on category of death was not available in DCIPS, it was obtained from the Department of Defense (DOD) Medical Mortality Registry maintained by the Mortality Surveillance Division of the Armed Forces Medical Examiner System (AFMES). Category of death, determined by a civilian or AFMES coroner or medical examiner, was categorized as either accidental (i.e., unintentional injury), natural, suicide, homicide, combat (separate from homicide), undetermined, or pending (separate from undetermined). Combat and pending deaths are not consistent with National Association of Medical Examiner standards and guidelines of 5 “manners of death,” so the term “category of death” is used instead, as “manner of death” has a specific definition.^10^ Combat deaths occur in theater because of hostile actions. Deaths still under investigation are classified as pending but are typically reclassified within 12 months. Data from DCIPS and AFMES were obtained in November 2021.

For underlying causes of death, the Suicide Data Repository (SDR), created and maintained by the DOD and Veterans Affairs, served as the primary source of information, as this information is not available from the AFMES or DCIPS.^11^ These data were obtained in November 2020. Cause of death is defined as the event that initiated the sequence of events resulting in death, recoded from International Classification of Diseases, 10th Revision (ICD-10) codes obtained from the National Death Index.^12,13^ For example, if accident is a category of death, then possible causes of death could be drowning, poisoning, or falls. Causes are not presented for combat-related deaths, because this category is based on only 2 ICD-10 codes: Y36 (Operations of war) and Y89.1 (Sequelae of war operations); these definitions were obtained from the World Health Organization ICD-10 manual.^12^

Demographic characteristics such as sex (female, male) and age (17-24, 25-34, 35-44, 45-64 years) were obtained from the DCIPS. Race and ethnicity (non-Hispanic White, non-Hispanic Black, non-Hispanic Asian/Pacific Islander [A/PI], Hispanic, non-Hispanic American Indian/Alaskan Native, unknown) and Army population estimates were obtained from the Defense Manpower Data Center (DMDC). To obtain the total U.S. Army active duty population, each component's troop counts for September of each year were derived from DMDC.

**Analytical Approach**

Univariate statistics (counts, percentages) were used to report the distribution of the categories of death, stratified by cause, age, sex, and race and ethnicity, from 2014 to 2019. The 5 leading underlying causes of death were reported overall as well as stratified by age, sex, and race and ethnicity based on counts. Leading underlying causes of death refers to the 5 most frequently occurring causes with the largest number of deaths reported over the 6-year period.

Crude annual mortality rates by category of death from 2014 to 2019 were calculated by dividing the number of deaths by the number of soldiers per year, multiplied by 100,000. Annual rates for the combat and homicide deaths were not included due to the high number of instances with less than 20 cases.^14^ Rate ratios (RRs) and 95% confidence intervals (CIs) of trend analyses were calculated using Poisson regression. Mortality data are not subject to sampling error because it is expected that all deaths in the population are captured, so 95% CIs are not reported for crude rates.^15^ All data management and statistical analyses were conducted using SAS® (version 9.4, SAS Institute, Inc., 2013, Cary, NC).

## 3 RESULTS

**Category of Death**

Between 2014 and 2019, 2,530 deaths occurred among U.S. Army soldiers (**Table 1**). During this period, suicide (n=883, 35%) was the most common category of death, followed by accidental death (n=814, 33%). Gunshot wounds (GSWs) accounted for 65% of suicide deaths, and about two-thirds of accidental deaths were transportation-related (67%). Natural death (n=534, 21%), the next most frequent category, was often caused by neoplasms or cancer (49%). GSWs were the cause of 79% of homicide deaths, and if legal interventions (i.e., legal execution or death by law enforcement) are included, that number increases to 82%.

**Cause of Death**

Overall, the 5 leading causes of death from 2014 to 2019 were suicide by GSW (n=575), motor vehicle accidents (MVAs) (n=431), neoplasms (n=263), suicide by hanging or asphyxiation (n=228), and cardiovascular events (n=145). When stratified by age group, MVAs were the leading cause for soldiers aged 17-24 years (**Table 2**). Accidental overdose (AOD) and homicide by GSW were the fourth and fifth leading causes for soldiers under age 35. Neoplasms were the leading cause in the oldest age group and women. The leading cause of death in non-Hispanic Black soldiers was MVAs (n=100). AOD was the fifth leading cause of death for non-Hispanic White soldiers.

**Trends in Mortality Rates**

From 2014 to 2019, suicide was generally the category with the highest cumulative mortality rate, followed by accidental death (**Figure**), with the exception of 2017. The crude rate of accidental death showed a slight annual upward trend of 2% (RR=1.02, 95% CI: 0.99-1.06) as it increased from 24.7 deaths per 100,000 soldiers in 2014 to 26.3 deaths per 100,000 soldiers in 2019 (**Table 3**). The annual rate of suicide death also showed a slight upward trend of 3% (RR=1.03, 95% CI: 1.00-1.07), as it increased from 25.4 deaths per 100,000 soldiers in 2014 to 28.8 deaths per 100,000 soldiers in 2019. Neither of these trends was statistically significant, however. The mortality rate for natural causes declined 6% (RR=0.94, 95% CI: 0.89-0.98) annually, from 18.8 deaths per 100,000 soldiers in 2014 to 13.4 deaths per 100,000 soldiers in 2019, which was statistically significant.

## 4 DISCUSSION

This is the first report since 2016 to expand on the underlying leading causes of death stratified by each demographic characteristic in the U.S. Army. The highest mortality rates were for suicide, and suicide by GSW remained the leading cause of death. The Army implements various initiatives that evaluate, identify, and track high-risk individuals for suicidal behavior and other adverse outcomes.^16,17^ Current measures are used to track and educate soldiers on securing privately-owned weapons—as the literature has concluded that storing firearms locked, unloaded, or both are associated with a lower risk of suicide mortality—but findings on the effectiveness of these programs are limited.^18,19^ A more passive approach, such as strict gun control policies, should also be considered.^19,20,21^ For instance, in a report released in 2023 by the Suicide Prevention and Response Independent Review Committee (SPRIRC) recommendations included establishing and updating gun control and safety policies to include requiring all privately-owned weapons in DOD military property to be registered and properly stored, and implementing waiting periods and minimum age requirements for privately-owned weapons and ammunition purchases on DOD property.^22^

Accidental death was the next most frequent category of mortality. Although no significant trend was detected in this study, the rate has decreased substantially since 2011.^23,24^ MVAs were the second leading cause of death overall, and for the youngest age group as well as non-Hispanic Black soldiers. Prior studies have suggested this may be due to inexperience, high rates of alcohol use, and lower likelihood of wearing seatbelts increasing odds of death.^25,26^ The United States Army Combat Readiness Safety Center’s mass safety campaigns aim to reduce transportation-related crashes, but programs tailored to these high-risk groups may be necessary to affect change.^27^ AOD was the fifth leading cause of death for non-Hispanic White service members, as well as the fourth leading cause for soldiers under age 35, which aligns with findings from prior reports that demonstrated higher rates of substance abuse and dependence among these groups.^28^

During this same period, the mortality rate for natural deaths declined significantly. Similar decreasing trends in deaths from natural causes such as heart disease and cancer were observed within the U.S. population from 2018 to 2019.^29,30^ Neoplasms are still the leading cause of death for female and older soldiers, and among women this result may be related to low cancer screening rates, based on findings in the literature. Recent studies have concluded that female service members were not compliant with breast or cervical cancer screening guidelines despite universal access to health care and completion of the Periodic Health Assessment (PHA) every 13 months.^31,32,33,34^ The PHA tracks cancer screening for breast, cervical, and colorectal cancers, as well as risk factors for lung cancer (e.g., smoking and tobacco use). Cardiovascular diseases and events were also a leading natural cause of death. This may be related to several cardiovascular risk factors observed in soldiers such as high blood pressure, smoking, and high body mass index.^35^ To improve the health and well-being of its service members, the DOD has implemented initiatives such as the Performance Triad (P3), which establishes guidelines for increasing physical activity, eating a well-balanced diet, and receiving adequate sleep, and which have shown to be protective against adverse health outcomes in service members.^36,37^

Due to the 2-year data lag in mortality data, the number of cases missing underlying causes of death was highest in 2019. As a result, reporting for that year may underestimate the true mortality burden. Active duty soldiers who separated from the Army were excluded, thereby underestimating a soldier’s risk of death, as previous studies have found higher mortality rates among separated soldiers compared to those who did not.^38^ Small sample sizes were an issue for some subgroups, particularly American Indian/Alaskan Natives, and findings for this group should be interpreted with caution. Furthermore, population estimates for September of each year were used to calculate rates, which may have led to inaccurate estimates. Despite these limitations, these data are comprehensive and capture all deaths among active duty soldiers while in service during the surveillance period.

From 2014 to 2019, when annual mortality rates for natural deaths significantly declined, the highest Army mortality rates were for suicide, followed by accidental death. Evaluation of various public health suicide prevention programs and services, and a greater emphasis on firearm storage and safety, may be needed to reduce suicide. Public health campaigns promoting safe driving habits and healthy behaviors can be refined by examining a combination of the underlying causes of death and contributing factors that provide contextual information for developing effective targeted prevention efforts. Despite the decline in natural deaths, neoplasms remain the leading cause of death in women and older soldiers, underscoring the importance of promoting healthy behaviors and staying up-to-date with cancer screenings.

## Figures and Tables

**Table 1:** Categories^a^ and Causes^b^ of Death Among U.S. Army Active Duty Soldiers^c^,2014–2019 (n=2,530)^d^

	Year of Death, n (%)						
	2014	2015	2016	2017	2018	2019	2014-2019
	(n=434)	(n=415)	(n=385)	(n=431)	(n=427)	(n=419)	(n=2,511)
Combat	**31 (7)**	**31 (1)**	**12 (3)**	**20 (5)**	**14 (3)**	**16 (4)**	**96 (4)**
Accident^e^	**136 (31)**	**137 (33)**	**123 (32)**	**154 (36)**	**128 (30)**	**136 (32)**	**814 (32)**
Motor vehicle	76 (56)	71 (52)	70 (57)	77 (50)	66 (52)	71 (52)	431 (53)
Motorcycle	15 (11)	13 (10)	12 (10)	10 (7)	11 (9)	2 (1)	63 (8)
Air, space, other transportation^f^	9 (7)	12 (9)	3 (2)	10 (7)	7 (5)	6 (4)	47 (6)
Drug/alcohol overdose^g^	16 (12)	21 (15)	18 (15)	31 (20)	24 (19)	11 (8)	121 (15)
Drowning^h^	6 (4)	7 (5)	9 (7)	9 (6)	5 (4)	12 (9)	48 (6)
Fall^i^	5 (4)	5 (4)	2 (2)	3 (2)	3 (2)	5 (4)	23 (3)
Other^j^	6 (4)	5 (4)	9 (7)	11 (7)	8 (6)	10 (7)	49 (6)
Unknown^k^	3 (2)	3 (2)	0	3 (2)	4 (3)	19 (14)	32 (4)
Natural^l^	**104 (24)**	**109 (26)**	**85 (22)**	**84 (20)**	**84 (20)**	**68 (16)**	**534 (21)**
Neoplasm	56 (54)	58 (53)	48 (57)	38 (45)	34 (40)	29 (43)	263 (49)
Circulatory system	30 (29)	36 (33)	19 (22)	23 (27)	30 (36)	7 (10)	145 (27)
Other^m^	17 (16)	11 (10)	12 (14)	11 (13)	11 (13)	8 (12)	70 (13)
Unknown^k^	1 (1)	4 (4)	6 (7)	12 (15)	9 (11)	24 (35)	56 (11)
Suicide^n^	**140 (32)**	**144 (35)**	**144 (37)**	**139 (32)**	**165 (39)**	**151 (36)**	**883 (35)**
Gunshot wound	99 (71)	92 (64)	99 (69)	95 (68)	103 (62)	87 (58)	575 (65)
Hanging/asphyxiation	28 (20)	38 (26)	34 (24)	36 (26)	44 (27)	48 (32)	228 (26)
Drug/alcohol overdose^g^	7 (5)	8 (6)	5 (4)	6 (4)	6 (4)	1 (1)	33 (4)
Other^o^	5 (4)	5 (3)	3 (2)	2 (1)	7 (4)	3 (2)	25 (3)
Unknown^k^	1 (1)	1 (1)	3 (2)	0	5 (3)	12 (8)	22 (2)
Homicide	**17 (4)**	**14 (3)**	**16 (4)**	**17 (4)**	**17 (4)**	**13 (3)**	**94 (4)**
Gunshot wound	11 (65)	12 (86)	9 (56)	16 (94)	15 (88)	11 (85)	74 (79)
Sharp object	3 (18)	2 (14)	4 (25)	0	1 (6)	2 (15)	12 (13)
Legal intervention^p^	2 (12)	0	1 (6)	0	0	0	3 (3)
Other^q^	0	0	1 (6)	1 (6)	1 (6)	0	3 (3)
Unknown^k^	1 (6)	0	1 (6)	0	0	0	2 (2)
Undetermined^r^	**5 (1)**	**8 (2)**	**5 (1)**	**15 (3)**	**5 (1)**	**3 (1)**	**41 (2)**
Pending^s^	**1 (<1)**	**0**	**0**	**2 (<1)**	**14 (3)**	**32 (8)**	**49 (2)**

Abbreviation: n, number.

^a^Category of death was obtained from the Defense Casualty Information Processing System or the Armed Forces Medical Examiner System.

^b^Underlying cause of death was obtained from the Suicide Data Repository (SDR), maintained by the DOD and Veterans Affairs.

^c^Includes Army active component, activated National Guard, and activated Reserve soldiers. Due to rounding, some percentages may add up to more than 100.

^d^Total includes all 19 deaths with missing category or cause information.

^e^Excludes 8 deaths that were missing cause of death information.

^f^Other transportation includes rail, water transport, and all other transportation.

^g^Drug/alcohol overdose includes poisonings from other solids and liquids, including medications.

^h^Includes accidental drowning in any body of water.

^i^Includes falls from high places, ladders, and any other type of fall.

^j^Includes explosions, pending, and all other accidental deaths.

^k^Includes any deaths that have no known cause of death or are classified as unknown.

^l^Excludes 5 deaths missing cause of death information.

^m^Includes diseases related to nervous system, respiratory system, digestive system, musculoskeletal system, mental and behavioral disorders, congenital malformations, blood, endocrine, skin, pregnancy, infections, surgical complications, and all other natural conditions.

^n^Excludes 3 deaths missing cause of death information.

^o^Includes carbon monoxide and other gas/vapor poisonings, jumping from a high place, and all other means.

^p^Legal intervention includes legal execution and deaths by police or other law enforcement agents.

^q^Includes strangulation, blunt object, bodily force, and all other means.

^r^Excludes 2 deaths missing cause of death information.

^s^Excludes 1 death missing cause of death information.

**Table 2:** Leading Underlying Causes^a^ of Death Among U.S. Army Active Duty Soldiers, 2014–2019 (n=2,530)

Subgroup^b^	Cause of Death^c^	Death Count (n)
Sex		
Male	Suicide by gunshot wound	538
	Motor vehicle accident^d^	401
	Suicide by hanging/asphyxiation	214
	Neoplasm^e^	211
	Cardiovascular disease and events^f^	130
Female	Neoplasm^e^	52
	Suicide by gunshot wound	37
	Motor vehicle accident^d^	30
	Cardiovascular disease and events^f^	16
	Suicide by hanging/asphyxiation	14
Age, y		
17-24	Motor vehicle accident^d^	215
	Suicide by gunshot wound	195
	Suicide by hanging/asphyxiation	106
	Accidental overdose	44
	Homocide by gunshot wound	34
25-34	Suicide by gunshot wound	245
	Motor vehicle accident^d^	152
	Suicide by hanging/asphyxiation	74
	Accidental overdose	48
	Homocide by gunshot wound	31
35-44	Suicide by gunshot wound	108
	Neoplasm^e^	91
	Cardiovascular disease and events^f^	60
	Motor vehicle accident^d^	51
	Suicide by hanging/asphyxiation	42
45-64	Neoplasm^e^	106
	Cardiovascular disease and events^f^	41
	Suicide by gunshot wound	27
	Other illness^g^	22
	Motor vehicle accident^d^	19
Race and ethnicity		
White, non-Hispanic	Suicide by gunshot wound	383
	Motor vehicle accident^d^	252
	Neoplasm^e^	162
	Suicide by hanging/asphyxiation	145
	Accidental overdose	98
Black, non-Hispanic	Motor vehicle accident^d^	100
	Suicide by gunshot wound	85
	Neoplasm^e^	63
	Cardiovascular disease and events^f^	42
	Suicide by hanging/asphyxiation	27
Hispanic	Suicide by gunshot wound	64
	Motor vehicle accident^d^	50
	Suicide by hanging/asphyxiation	33
	Neoplasm^e^	21
	Cardiovascular disease and events^f^	17
Asian/Pacific Islander, non-Hispanic	Suicide by gunshot wound	19
	Suicide by hanging/asphyxiation	16
	Neoplasm^e^	12
	Motor vehicle accident^d^	10
	Accidental drowning	5
American Indian/Alaskan Native, non-Hispanic	Suicide by gunshot wound	6
	Motor vehicle accident^d^	6
	Suicide by hanging/asphyxiation	2
	Homocide by gunshot wound	2
	Accidental overdose	1

Abbreviations: n, number; y, years.

^a^Cause of death based on the ICD-10 National Center for Health Statistics records and obtained from the Suicide Data Repository.

^b^Information on demographic characteristics was obtained from the Defense Casualty Information Processing System or Defense Manpower Data Center.

^c^Rank based on number of deaths.

^d^Includes accidents involving heavy transport vehicles, buses, and individuals injured in collisions with motor vehicles, regardless of whether passenger, driver, or pedestrian.

^e^Includes deaths directly attributed to primary or secondary neoplasms and complications of neoplasms.

^f^Includes cardiac events, embolisms, aneurysms, strokes, and hemorrhages.

^g^Includes diseases related to nervous system, respiratory system, digestive system, musculoskeletal system, mental and behavioral disorders, congenital malformations, blood, endocrine, skin, pregnancy, infections, surgical complications, and all other natural conditions.

**Figure:**
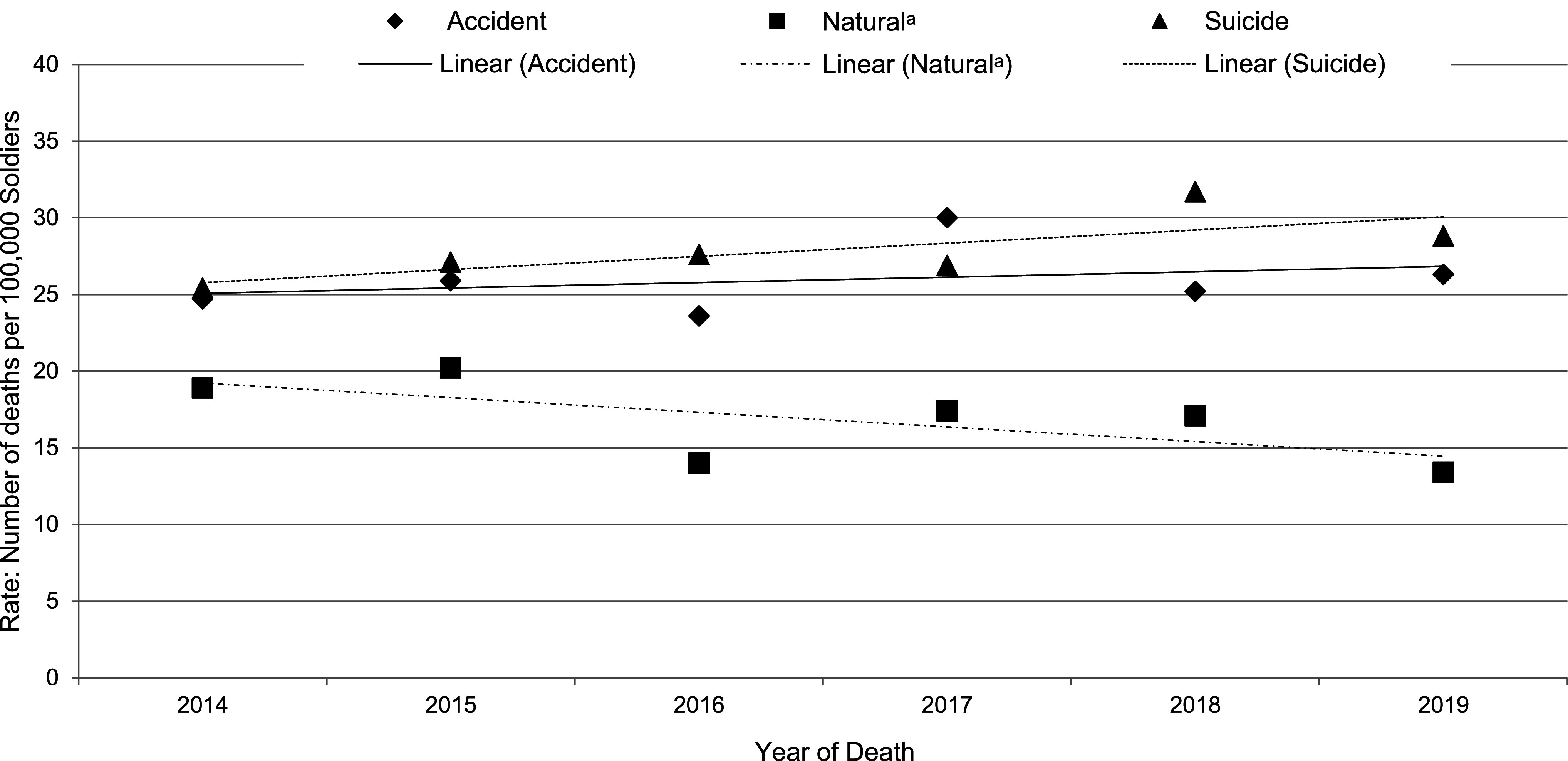
Annual Crude Mortality Rates by Category of Death Among U.S. Army Active Duty Soldiers, 2014–2019

**Table 3:** Annual Crude Mortality Rates^a^ for U.S. Army, 2014–2019

	Category of Death^b^		
Year of Death	Accident	Natural	Suicide
2014	24.7	18.9	25.4
2015	25.9	20.2	27.1
2016	23.6	14.0	27.6
2017	30.0	17.4	26.9
2018	25.2	17.1	31.7
2019	26.3	13.4	28.8
Rate Ratio^c^ (95% CI^d^)	1.02 (0.99–1.06)	**0.94 (0.89–0.98)**	1.03 (1.00–1.07)

Abbreviation: CI, confidence interval.

^a^Mortality rates are interpreted as the number of deaths per 100,000 soldiers. Numerator data were obtained from the Defense Casualty Information Processing System and the Armed Forces Medical Examiner System; denominator data were obtained from the Defense Manpower Data Center.

^b^Combat and, homicide death rates are not presented due to less than 20 deaths for all or most years.

^c^Rate ratios, calculated using Poisson regression, assessed trends in mortality rates from 2014 to 2019, bolded numbers are statistically significant.

## AUTHOR AFFILIATIONS

General Dynamics Information Technology Inc., Falls Church, VA: Ms. Kaplansky; Division of Behavioral and Social Health Outcomes Practice, Defense Centers for Public Health–Aberdeen, Defense Health Agency, Aberdeen Proving Ground, MD: Ms. Kaplansky, Dr. Toussaint
